# Postoperative Tongue Exercises for Ankyloglossia Following Lingual Frenectomy: A Case Report

**DOI:** 10.7759/cureus.69806

**Published:** 2024-09-20

**Authors:** Aditi A Dodal, Anup U Shelke, Chitrika Subhadarsanee, Subodh P Gaikwad, Kuldeep S Patil, Pavan Bajaj

**Affiliations:** 1 Department of Periodontology, Dr. Hedgewar Smruti Rugna Seva Mandal's (HSRSM) Dental College and Hospital, Hingoli, IND; 2 Department of Periodontics, Sharad Pawar Dental College and Hospital, Datta Meghe Institute of Higher Education and Research, Wardha, IND

**Keywords:** ankyloglossia, electrosurgery, lingual frenectomy, postoperative tongue exercises, tongue-tie

## Abstract

Ankyloglossia, also named tongue-tie, is an innate developmental anomaly where the lingual frenum appears anchored to the sublingual space. Even though it is not considered a major congenital condition, it can create a variety of problems, such as difficulty in feeding newborns, difficulty in mastication, speech problems, poor oral hygiene, malocclusion, and hindrance in social interaction because of restricted tongue movements. Lingual frenectomy is the treatment of choice. Postoperative tongue exercises should be considered a valuable addition after lingual frenectomy, providing the patient with enhanced free tongue movement along with kinesthetic awareness of the tongue.

## Introduction

In 1963, the term "ankyloglossia" was first introduced in the literature by Wallace as a condition where the tongue tip is unable to protrude beyond the lower incisor teeth because of a short lingual frenum, which is present with a scar tissue most of the time [1]. The small tissue strip connects the tongue's ventral surface to the mouth floor vertically. Ankyloglossia may occur because of the frenum's tight, thick structure or from its location of insertion restricting the tongue's range of movement [2].

Kotlow, in 1999, classified ankyloglossia based on the length of the free tongue into four categories. "Free tongue" is the tongue length of the lingual frenum from its insertion into the tongue base to the tongue tip. More than 16 mm of free tongue length is considered normal and accepted clinically. About 12-16 mm of free tongue length is class I, mild ankyloglossia; 8-11 mm is class II, moderate ankyloglossia; 3-7 mm is class III, severe ankyloglossia; and less than 3 mm of free tongue length is class IV, complete ankyloglossia [3].

Various problems, such as difficulty in feeding newborns, difficulty in mastication, speech problems, and malocclusion in children, are encountered because of ankyloglossia. However young adults and adolescents face hindrances in academic activities and social interaction because of restrictions on tongue movements [4]. Speech difficulties associated with ankyloglossia include difficulty pronouncing the sounds s, r, j, ch, and zh as well as fluent speech in general. The growth of the upper airway and maxillofacial complex may be impacted by ankyloglossia. Other potential effects of limited tongue mobility include snoring and mouth breathing [5].

Periodontal health may be compromised by ankyloglossia. Because of its ability to self-clean, the tongue's regular range of motion makes it a great tool for maintaining oral hygiene. Ankyloglossia limits the tongue's ability to brush against food particles. Recession of the gingiva in the mandibular anterior lingual region can result from ankyloglossia. In most of the cases, ankyloglossia remains asymptomatic [6].

Lingual frenectomy is the choice of treatment for ankyloglossia. Comparing different methods for frenectomy, electrosurgery is advantageous over laser as it is less expensive and over conventional because it causes no bleeding, requires no suturing, and is relatively less painful with less discomfort [2]. Frenectomy alone might not yield the desired effects, particularly in adults and teenagers, where neuromuscular re-education is necessary for the tongue. Postoperative tongue exercises are the best way to regain this awareness that is often lacking in surgically treated ankyloglossia cases [7].

In this report, we present two cases: a 23-year-old male and a 17-year-old female. Both were diagnosed with ankyloglossia and treated with lingual frenectomy using electrosurgery and adjunctive tongue exercises postoperatively.

## Case presentation

Case 1

**Figure 1:**
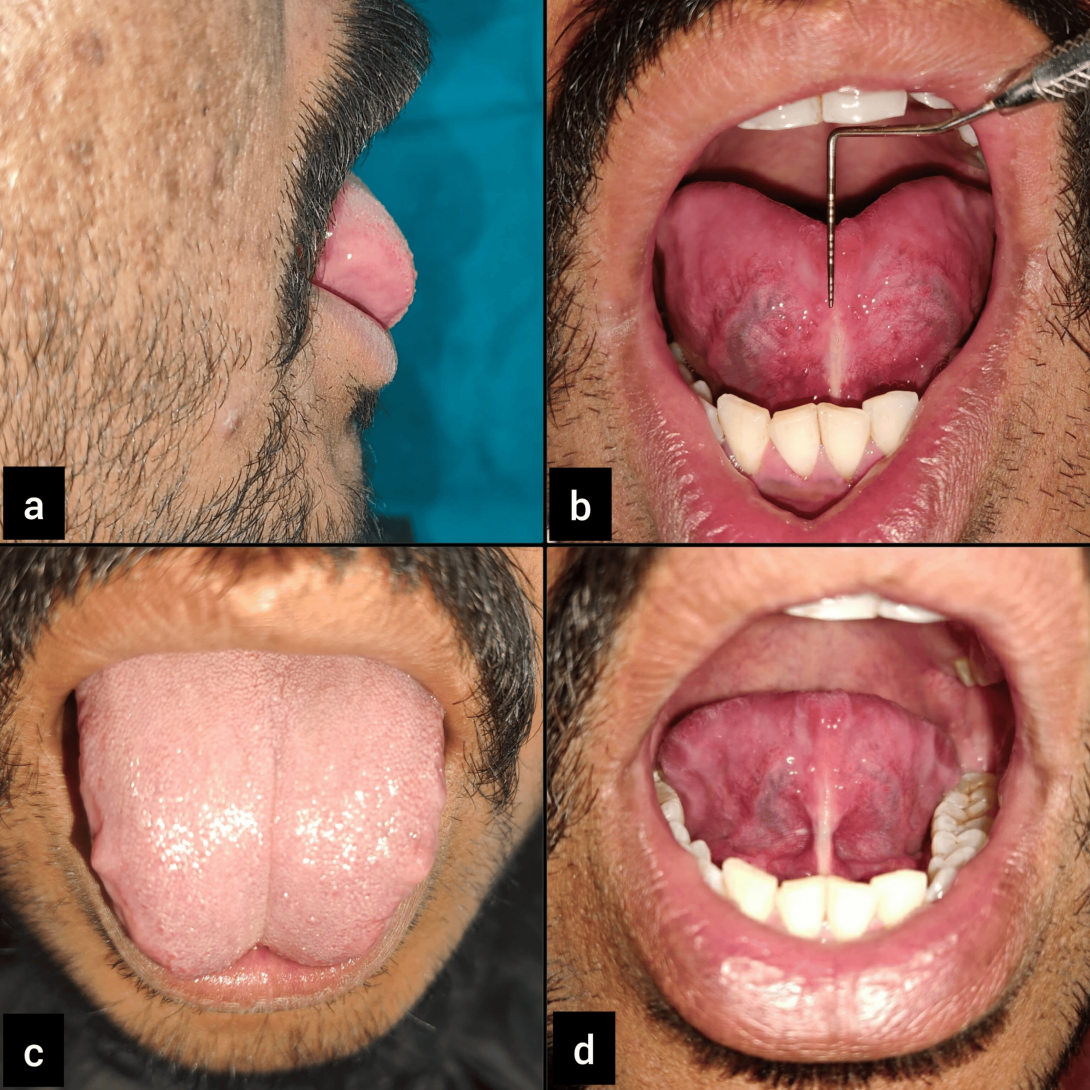
Case 1: (a) preoperative view; (b) 10 mm of free tongue length; (c) on protrusion, the tongue represents "W"-shaped appearance, a classic feature of ankyloglossia; and (d) the inability of the tip of the tongue to curl toward the palate.

A 23-year-old male patient reported to the Department of Periodontics, with a complaint of difficulty in pronouncing words with s, r, and ch sounds. The findings were recorded through general and oral examinations as well as a speech assessment, which revealed a 10 mm free tongue length, indicating moderate ankyloglossia, classified as Kotlow’s class II type (Figure 1, Panel b).

Case 2

**Figure 2:**
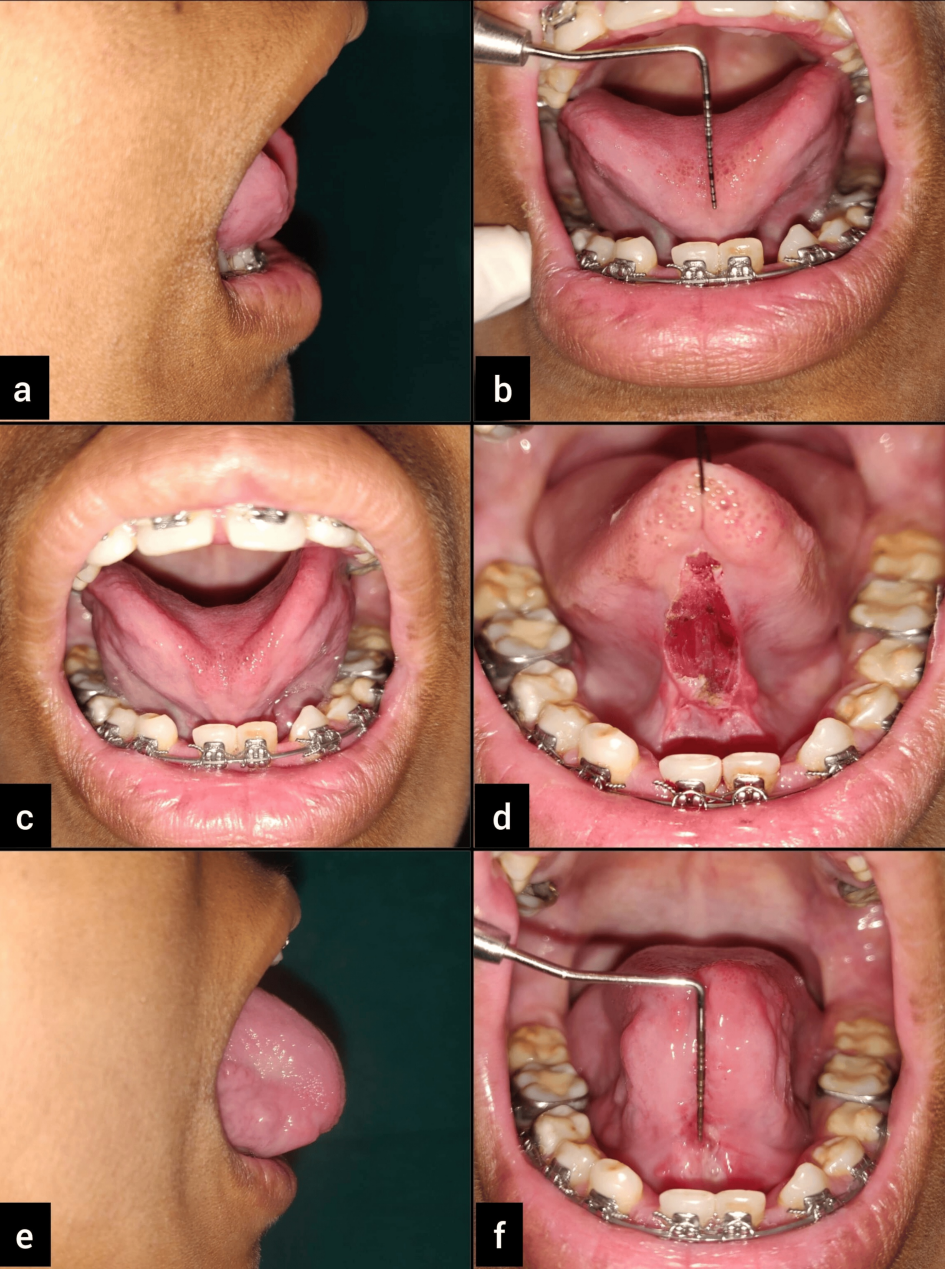
Case 2: (a) preoperative view; (b) 6 mm of free tongue length; (c) the inability of the tip of the tongue to curl toward the palate; (d) immediate postoperative view showing arrested bleeding and no need for sutures; (e) three months post-surgery; and (f) more than 16 mm of free tongue length achieved after tongue exercises.

A 23-year-old female patient reported to the Department of Periodontics, with a complaint of difficulty in the pronunciation of words with r and zh sounds. The findings were recorded through general and oral examinations as well as a speech assessment, which revealed 6 mm of free tongue length, indicating severe ankyloglossia, classified as Kotlow’s class III type (Figure 2, Panel b).

In both cases, the tongue tip on protrusion represented a "W"-like appearance, which is a classic feature of ankyloglossia (Figure 1c). The diagnosis was made as Kotlow’s class II ankyloglossia for Case 1 and Kotlow’s class III ankyloglossia for Case 2. Lingual frenectomy was advised as a treatment for relieving the frenum, allowing normal tongue movements. This was followed by postoperative exercises to improve awareness kinesthetically so that the tongue can achieve an entire range of movements.

**Figure 3:**
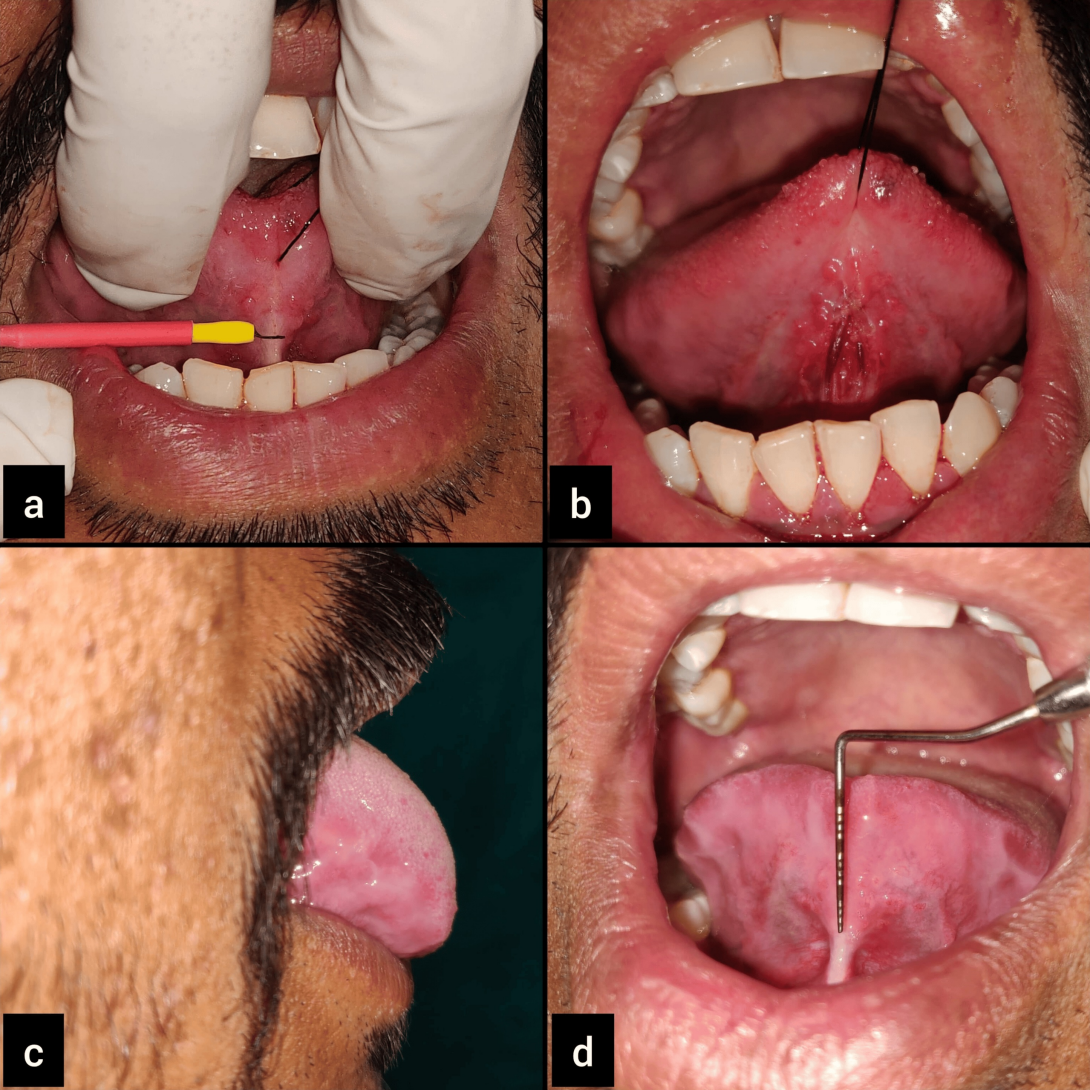
Case 1: (a) excision of lingual frenum with electrosurgery unit; (b) immediate postoperative view showing arrested bleeding and no need for sutures; (c) three months post-surgery; and (d) more than 16 mm of free tongue length achieved after tongue exercises.

The patients were informed about the treatment plan and consent was obtained for the same. The area was anesthetized using infiltration of 2% lignocaine with 1:80,000 adrenaline. The excision of the lingual frenum was made using the electrosurgery unit (Figure 3, Panel a). Using gentle brushing strokes, incisions were made with a scalpel point electrode. All the fiber remnants were excised, and blunt dissection was performed. With a wet gauze piece, the burned tissue was constantly wiped to clean up and avoid more heat damage to the soft tissue underneath.

Bleeding was almost negligible during and after the procedure, with minimal postoperative discomfort and pain (Figure 2d, 3b). Post-surgical instructions were provided. Analgesics (paracetamol tablets of 500 mg) were prescribed to the patient and asked to be taken if needed. A follow-up appointment was scheduled after one week, and satisfactory wound healing was observed.

Postoperative tongue exercises

**Figure 4:**
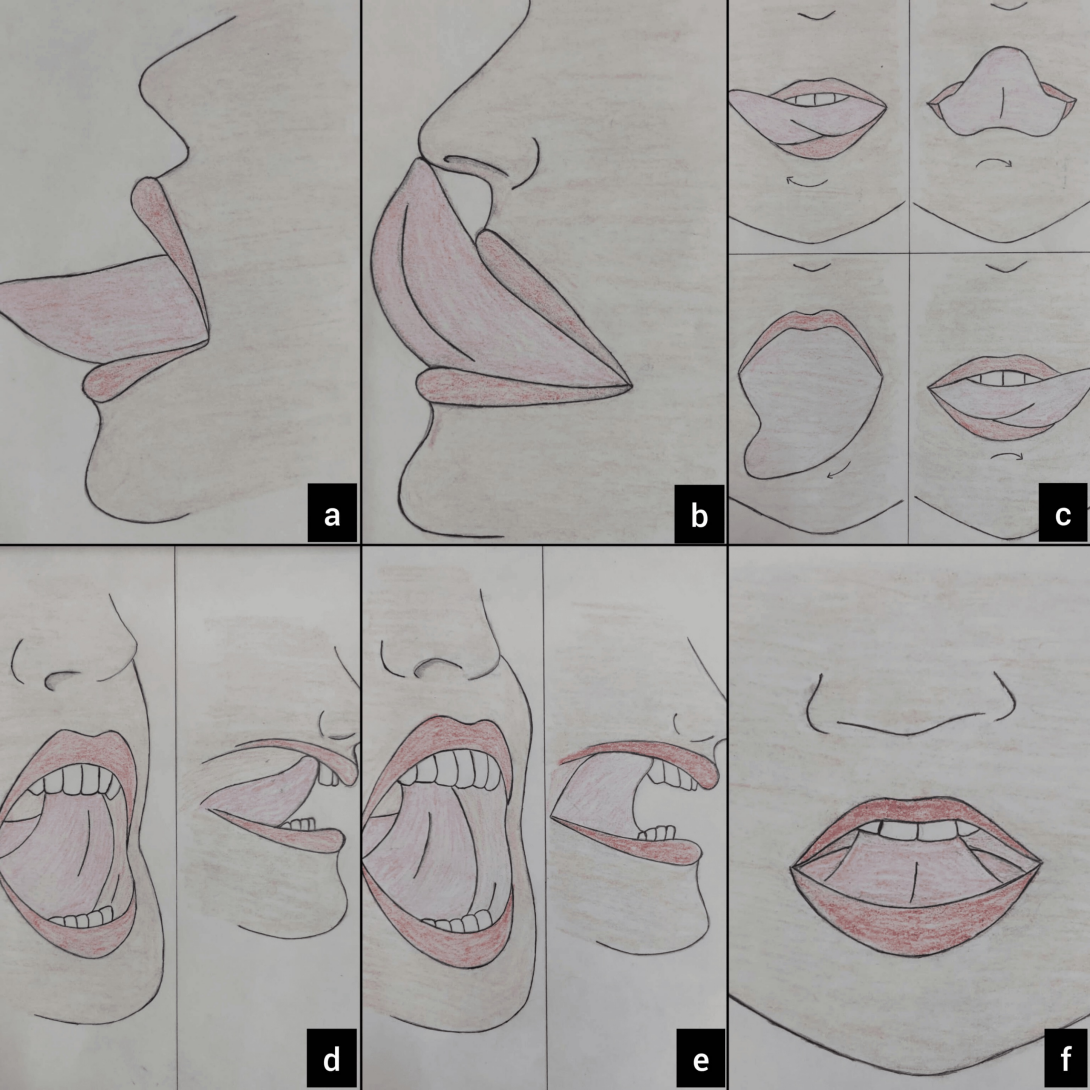
(a) Tongue protrusion to its maximum extent; (b) protrusion of the tongue to its maximum extent with tongue movement trying to touch the nose tip; (c) protrusion of the tongue to its maximum extent and circular rotatory movements, in clockwise and anti-clockwise directions; (d) wide opening and closing of mouth with the tongue tip positioned on the incisive papilla; (e) wide opening and closing of mouth with the tongue tip positioned on the middle of the palate and the posterior palate; and (f) the mouth is gently expanded to its utmost extent until the tongue loses touch with the palate when the tongue is pressed against the palate completely and a vacuum is created by sucking the air. Image credits: Authors of this study. The figures are under the process of copyright with diary no.: 4113/2024-CO/L.

The protocol for postoperative tongue exercises recommended by Tecco et al. [7] and a pictorial depiction of tongue movements after the treatment of ankyloglossia (Figure 4) were described to the patients, and their cooperation was ensured.

**Figure 5:**
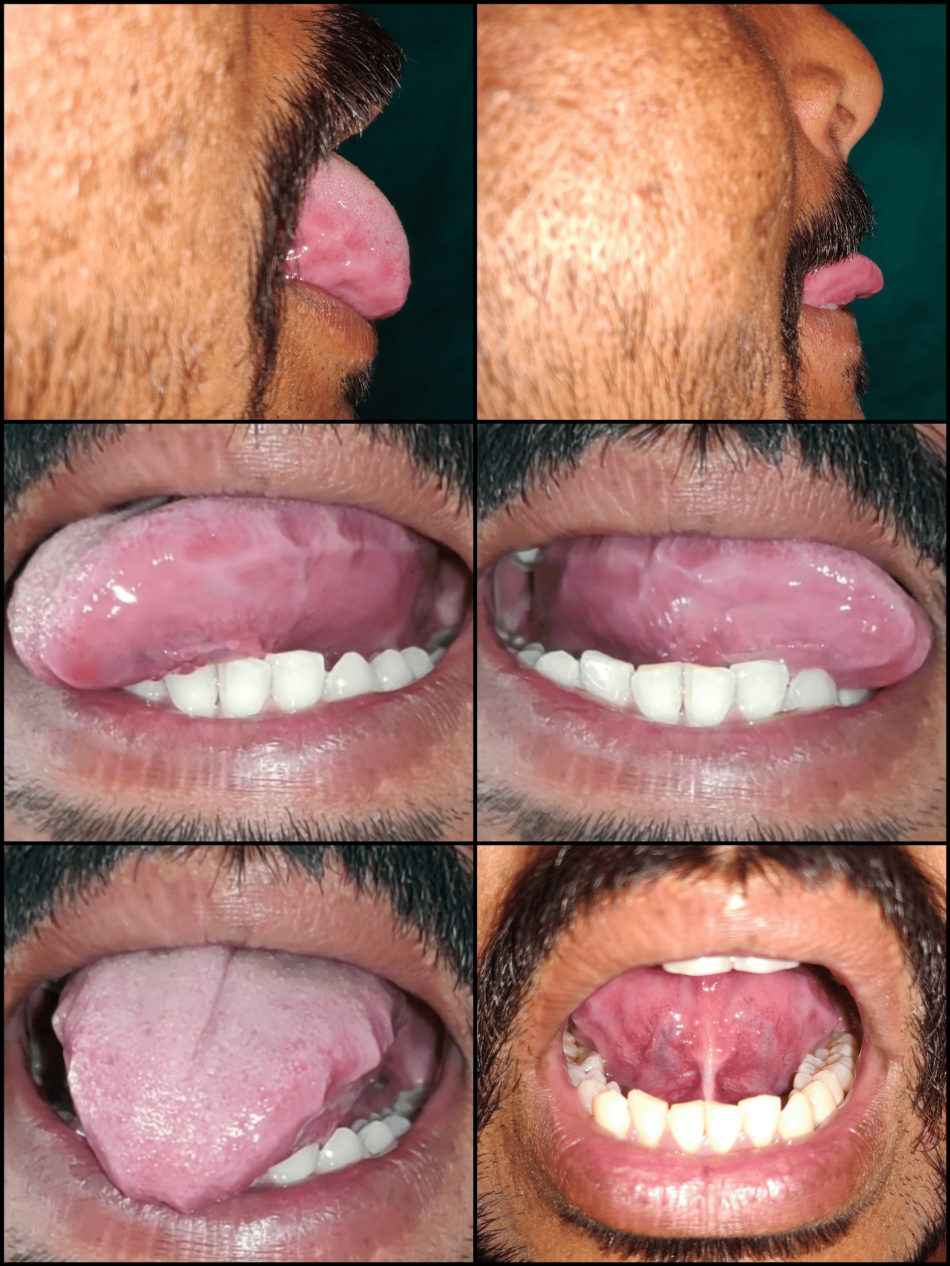
Case 1: depiction of postoperative tongue exercises

**Figure 6:**
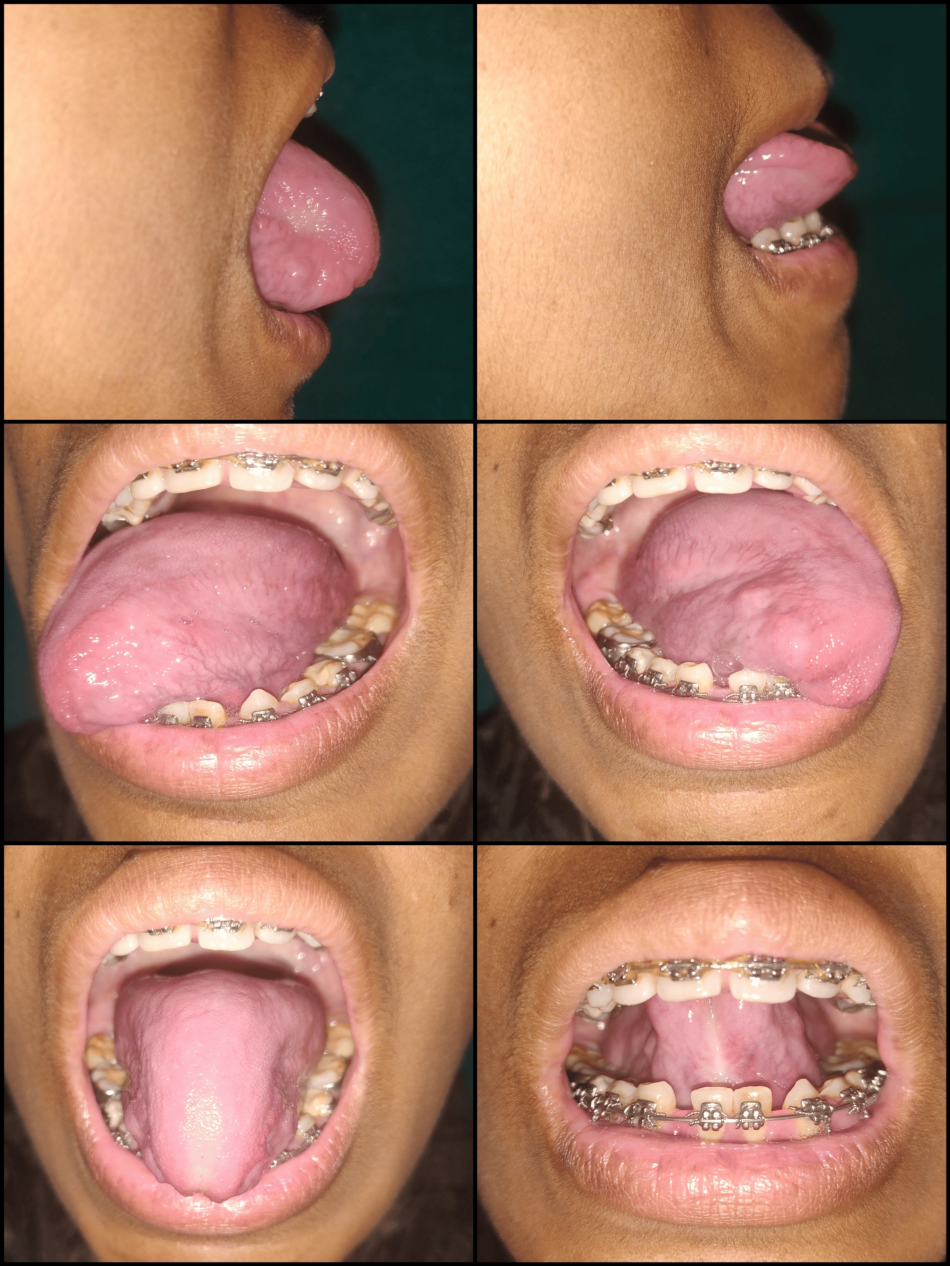
Case 2: depiction of postoperative tongue exercises

For improved compliance, the patient was instructed to exercise in front of a mirror 15 times, thrice a day, for three months (Figures 5, 6).

An appreciable increase in the mobility of the tongue was noted on review after three months (Figure 2f, 3d). The patient was more comfortable and confident while pronouncing words with the sounds r, s, z, ch, and zh.

## Discussion

Surgical methods such as frenectomy, frenotomy, or frenuloplasty are typically used to relieve the aberrant frenal attachment in cases of ankyloglossia. A complete excision and removal of the whole frenum is known as a frenectomy [8].

Hemostasis is challenging to attain during conventional scalpel surgery, with poor surgical site visibility and a higher risk of damaging important tissues. Due to the more invasive nature of frenectomy surgery, which involves blood loss, a large surgical cut, and suturing, patients undergoing conventional frenectomy treatments with a scalpel frequently experience pain and discomfort following the procedure. In contrast, electrosurgery provides the benefits of less time consumption, decreased fatigue for the operators, and hemostasis while cutting without the need for sutures. Reduced bleeding throughout the process facilitates better tissue manipulation and improves visibility to the surgical site. Sutures can add to postoperative discomfort as they obstruct normal processes like speech and food intake. Using electrosurgery to treat the patient eliminated the need for suturing and decreased the chance of a postoperative infection [2].

Comparing the benefits of frenectomy with laser and electrosurgery discovered that the former is similar to the latter. However, the primary drawback of a laser is its significantly higher cost compared to electrosurgical equipment [9]. According to reports, electrosurgery units perform incisions and excisions more quickly than lasers in terms of seconds. There is also reduced collateral tissue damage in the electrosurgery group compared to the laser group [2,10]. Other advantages of electrosurgery over laser are that no safety glasses are required and large amounts of tissue can be quickly removed [10]. One of the drawbacks of electrosurgery is its unpleasant smell. Studies comparing the healing after the excision of soft tissues using electrosurgery and a scalpel demonstrated that during electrosurgical procedures, lateral heat is generated which affects the wound healing adversely, thus delaying the healing process [10,11].

Tongue exercise is the next step in the thorough management of ankyloglossia, after prompt and suitable surgical treatment. The adjunctive kinesthetic therapy improved the patient's capacity to lift the tongue [8]. The importance of tongue exercises after surgery in the treatment of ankyloglossia is often underrated. The goal of postoperative exercises is to increase the tongue's kinesthetic awareness rather than to improve speech. The stomatognathic system, including various muscles of the oral cavity, partially compensates for the reduced function of the tongue caused by ankyloglossia. Consequently, following surgical repair of ankyloglossia, the "muscle memory" of the tongue is lost for its ideal, unrestricted function. This is when rehabilitative tongue exercises, together with orofacial myofunctional therapy, come in handy for retraining the tongue to restore its lost muscle memory [7].

In these cases, improvement was noticed in tongue mobility one week after surgery, though it was limited. Once tongue exercises were started, there was a noticeable improvement in tongue mobility. Three months postoperatively, the length of the free tongue was more than 16 mm. The patients experienced improved comfort and ease when performing various tongue movements.

## Conclusions

Management of ankyloglossia with lingual frenectomy and adjunctive postoperative tongue exercises is shown to yield improved and desired outcomes. This approach is particularly useful when treating teenagers and young adults, as it becomes crucial to retrain the tongue to use the lost muscle memory. With minimal pain and discomfort following electrosurgery-assisted frenectomy, the patient felt both psychologically and physically prepared to resume their exercises one week after surgery. The patient's compliance is necessary for better results, and speech therapy can be incorporated if needed.
